# Supplementary material for: Changes to the sample design and weighting methods of a public health surveillance system to also include persons not receiving HIV medical care
Source: PLoS One. 2020 Dec 3;15(12):e0243351. doi: 10.1371/journal.pone.0243351 (PMC7714102; doi:10.1371/journal.pone.0243351)
Supplement: S1 Appendix — (DOCX) [file pone.0243351.s001.docx]

S1 Appendix. AUC Statistics by Method and Project Area.

|  | **Noncontact Models** | | | **Contacted Nonresponse Models** | | | **Overall Nonresponse Models** | | |
| --- | --- | --- | --- | --- | --- | --- | --- | --- | --- |
| **Project Area** | **Propensity*** | **Full Cell*** | **Reduced Cell**** | **Propensity*** | **Full Cell*** | **Reduced Cell**** | **Propensity*** | **Full Cell*** | **Reduced Cell**** |
| **National** | 0.734 | 0.730 | 0.720 | 0.603 | 0.600 | 0.587 | 0.699 | 0.698 | 0.689 |
| **California** | 0.726 | 0.713 | 0.689 | 0.526 | 0.526 | N/A | 0.685 | 0.649 | 0.615 |
| **Delaware** | 0.761 | 0.762 | 0.692 | N/A | 0.609 | 0.609 | 0.762 | 0.778 | 0.709 |
| **Florida** | 0.723 | 0.730 | 0.692 | 0.676 | 0.661 | 0.635 | 0.737 | 0.725 | 0.704 |
| **Georgia** | 0.773 | 0.770 | 0.715 | 0.708 | 0.708 | 0.586 | 0.725 | 0.723 | 0.621 |
| **Illinois** | 0.792 | 0.790 | 0.638 | N/A | N/A | N/A | 0.698 | 0.689 | 0.567 |
| **Indiana** | 0.750 | 0.732 | 0.711 | N/A | N/A | N/A | 0.735 | 0.717 | 0.691 |
| **Michigan** | 0.680 | 0.714 | 0.678 | 0.675 | 0.619 | 0.584 | 0.657 | 0.662 | 0.620 |
| **Mississippi** | 0.844 | 0.843 | 0.772 | 0.630 | 0.630 | 0.630 | 0.813 | 0.806 | 0.746 |
| **New Jersey** | 0.774 | 0.735 | 0.716 | N/A | 0.624 | N/A | 0.769 | 0.735 | 0.720 |
| **New York** | 0.754 | 0.777 | 0.684 | N/A | 0.614 | 0.614 | 0.740 | 0.706 | 0.654 |
| **North Carolina** | 0.717 | 0.721 | 0.677 | N/A | N/A | N/A | 0.696 | 0.692 | 0.647 |
| **Oregon** | 0.673 | 0.673 | 0.673 | 0.548 | 0.548 | 0.548 | 0.645 | 0.645 | 0.633 |
| **Pennsylvania** | 0.578 | 0.578 | 0.578 | N/A | N/A | N/A | N/A | N/A | N/A |
| **Texas** | 0.703 | 0.701 | 0.686 | N/A | N/A | N/A | 0.652 | 0.656 | N/A |
| **Virginia** | 0.717 | 0.715 | 0.687 | 0.677 | 0.677 | N/A | 0.712 | 0.715 | 0.678 |
| **Washington** | 0.697 | 0.708 | 0.669 | N/A | N/A | N/A | 0.623 | 0.648 | N/A |
| **Puerto Rico** | 0.835 | 0.829 | 0.808 | 0.559 | N/A | N/A | 0.776 | 0.776 | 0.753 |
| **Chicago** | 0.769 | 0.787 | 0.733 | 0.599 | 0.599 | 0.599 | 0.688 | 0.683 | 0.645 |
| **Houston** | 0.710 | 0.707 | 0.688 | 0.750 | 0.779 | 0.677 | 0.747 | 0.749 | 0.714 |
| **Los Angele** | 0.802 | 0.790 | 0.763 | N/A | N/A | N/A | 0.721 | 0.712 | 0.687 |
| **New York City** | 0.820 | 0.820 | 0.773 | 0.571 | 0.571 | 0.571 | 0.790 | 0.790 | 0.745 |
| **Philadelphia** | 0.802 | 0.789 | 0.740 | N/A | N/A | N/A | 0.772 | 0.760 | 0.709 |
| **San Francisco** | 0.741 | 0.724 | 0.680 | N/A | N/A | N/A | 0.627 | 0.626 | N/A |
| *** "N/A" indicates cells where there were no significant predictors based on the bivariate analysis. As a result no multivariate model was computed.** | | | | | | | | | |
| **** "N/A" indicates cells where there were no variables selected based on the selection criteria. As a result no reduced multivariate model was computed.** | | | | | | | | | |
