# Supplementary material for: Changes to the sample design and weighting methods of a public health surveillance system to also include persons not receiving HIV medical care
Source: PLoS One. 2020 Dec 3;15(12):e0243351. doi: 10.1371/journal.pone.0243351 (PMC7714102; doi:10.1371/journal.pone.0243351)
Supplement: Attachment — Submitted filename: review.docx [file pone.0243351.s002.docx]

This research aimed to describe methods used for computing survey weights, the approach chosen, and the benefits of using a dynamic surveillance registry as a sampling frame for adults living with HIV from the National HIV Surveillance System in the United States. While the authors have reported an excellent research topic, I believe that the following comments can be also helpful to consider in their revised version. What I believe this manuscript lacks are bare explanations of the methods used in the Methods section and bare presentation of the Results section. The authors should provide a detailed description and presentation of their findings.

Title:

I believe the title should show the area of this research. This is why I think we should have an “HIV” word somewhere like, “… in order to include persons not receiving HIV medical care”. As well, the title can be shorter. For example, “in order to include persons not receiving” can be written as “to also include persons not receiving”. The word “also” (or any other alternative) in this suggested title can reflect that the system had not included such individuals, but these should be also included along with those who receive HIV care.

Abstract:

When the authors claim that “weighting class adjustments and a single-stage nonresponse adjustment performed best,” they have to be more specific in what regard these methods turn to be the “best.” Or, “strongly associated with” does not reflect anything unless we see some outputs or even some key findings. Please avoid using such vague sentences and provide more evidence for these results.

The abstract does not seem to be a good place talking about how the results of this HIV case study might also have implications for “other disease registries”. This can be highlighted in the main body of the manuscript.

Introduction:

Paragraph 2:

a) In the second paragraph, second line: does not this sentence “Persons with undiagnosed HIV or diagnosed but receiving medical care” should not be looked like: “Persons with undiagnosed HIV or diagnosed but NOT receiving medical care.”?

b) In the same sentence, this sentence “account for most new HIV transmissions” should be supported by some quantitative data or statistics.

Paragraph3:

In this sentence, “with the number of AIDS cases,” did the author mean “HIV and AIDS cases” or only AIDS cases? Make sure that these two in the current era (and even the past) are (were) different. If they rewrite all HIV cases, this may include those individuals in AIDS phase, but the other way round does not include HIV cases. Caution with regard to the use of AIDS and HIV terms should be made throughout the text.

Paragraph 7 (line 98-99): “and eligibility criteria reflect these criteria” is not clear; reflect which criteria?

I believe the Introduction should have another paragraph, before the Materials and Methods, to address the main objectives of this research and highlight the gaps for the current sampling strategy. From the rest of the introduction section, we found that there were two main designs for sampling, one from 2005 to 2014, and the other from 2015 to the present. But, what has been as limitations or has not been considered very well in these two designs, especially the present one, should be highlighted in the last paragraph when objectives are also listed.

Material and Method section:

Is there any possibility to better demonstrate Table 1 with the real values or statistics obtained from this research on how a final weight was constructed? Or, the authors may consider showing these steps in an MS Excel file how to construct such weights.

I believe that the authors should explain the reason(s) for each weight in a clear way. For example, “design weight” is a way to deal with sampling error (i.e., happens when the selected sample does not fully and accurately reflect sampling frame); “nonresponse weight” is a way to deal with nonresponse error which id defined as … .

Design weight paragraph (line 114):

The authors stated that the first component of the weight (design weight) was calculated as the reciprocal of the probability of selection (meaning the number of individuals in the frame population represented by each sample). While this is true, the authors should pay attention to the fact that this is true for the time when weights are incorporated based on a simple random sampling approach (i.e., equal inclusion probability for each individual in the sample frame). However, this is not true when other sampling strategies such a stratified random sampling or clustered random sampling might be necessary. In HIV research, and even in the context of this research, clustering sampling (where health facilities might be considered as clusters) is more common. How would different sampling strategies be accounted in this weighting process?

The definition of nonresponse adjustment/weigh (i.e., the reciprocal of the probability of responding) without considering the mechanism of missingness is incomplete. The probability that one (i) responds to the survey given that they were sample (P_r|s_) is different when we assume missing completely at random, or missing at random (I think this is what the authors selected as their mechanism, but there are no clear justifications in the manuscript), or not missing at random. Such a mechanism should be explained clearly in this manuscript.

Nonresponse adjustment paragraph:

Comment on the logistic regression model: a) absolute log odds ratio (|log(OR)|) probably means the absolute value of beta estimate (log odds ratio) obtained from logistic regression. This should be clearer; b) in this sentence, “to choose the most significant predictors”, I believe “significant predictors” in the context of statistics and epidemiology refers to statistically significant, while the authors used beta coefficients to choose the “strongest predictors” as these beta coefficients were ranked. This should be clarified; c) to find the strongest predictors (if this is the case for what the authors did in this step), using the unstandardized format of the variable cannot identify these strong predictors. The standardized version of the included variables should be used to identify such strong predictors. This is another consideration in this step.

To enrich the results and methods section of this piece, I would strongly recommend the authors to provide their statistical models for both multivariable logistic regression and propensity weighting approach. These can be provided in both the formula and their software codes (syntaxes). Consider to explain and provide them in an APPENDIX.

I am wondering if how the authors incorporated the score obtained from propensity score approach in the weighting process? I mean, whether they just included the continuous measure of the score in the model, or they used a categorical format of the score, or whether they inverse-probability-censoring weighting (IPCW) approach? If IPCW was used, was it a unstabilized (i.e., 1/Pr(r|covariates)) or a stabilized (Pr(r)/Pr(r/covariates); where r refers to respondents). The stabilized approach has been commonly recommended to be used in case this is the approach for weighting process. The authors should clarify this process. In case they only used propensity score, they should also explain why they did not use stabilized IPCW.

Using a propensity based approach requires several key assumptions. How did the authors check and satisfy the assumption? For example, one key assumption of propensity approach is “Positivity” or “common support”, which is for each value of X (which is a vector of covariates), there is a positive probability of being both exposed and unexposed (in this manuscript, both responded and non-responded): 0 < Pr(r = 1|covariates) < 1. Another assumption is “conditional independence”; how the authors checked this assumption? “Correct model specification” is another assumption. “Unmeasured confounders” is another assumption.: The model is corrected, given unmeasured confounders. Therefore, a detailed explanation of these assumptions is definitely required.

Noncoverage adjustment paragraph:

a) How the noncoverage adjustment/weight were calculated? Was it based on the % of one stratum in the “target population” divided by % of the same stratum in the sample, or the % of one stratum in the “frame population” divided by % of the same stratum in the sample? While the total number of target population (both diagnosed and undiagnosed individuals living with HIV) is unknown (regardless the fact that they can be estimated), I believe this should be clarified in this section that the frame sample was used to calculate this weight;

b) There is a model-based approach (called Raking) using the totals of HIV diagnosed population (assuming that this is the sample frame) to create post-stratification weights so that the marginal values of a categorical variable add up to the totals. Given the efficiency of this approach in including many strata in the process of post-stratification adjustment, I am wondering why this approach had not been incorporated into the weighting process to better improve the novelty of the approach.

Line 192: stratified PPS design should be spelled out as PPS (probability proportional to size).

Variance estimation:

How the author defined non-certainty PSUs? As well, what about certainty PSUs? These two types of PSUs require more explanations.

Table 1 is not well-linked with the text in the methods section. For example, the formula provided for the nonresponse adjustment stage in Table 1 should be clearly and accurately explained in the text. what are those elements in the numerator and what are those in the denominator? Consider explaining all these elements in the text.

Again, I strongly recommend the authors to provide a detailed process of the sampling when explaining each section of the methods and subsequently, the results section. Considering to have an MS Excel file (as an APPENDIX) explaining each step in a practical way is highly recommended. This can help the authors absorb more audiences who can evaluate their great research work, and probably improve it in the future. This can help the manuscript be an applied paper such that researchers in other fields (non-HIV fields) can also consider this approach. In each step they provide information, a comparison of the approaches (the one the authors recommended and the previous approach of sampling) is also recommended.

Results:

The author should provide an N for their sample frame; 9,700 persons were sampled from what N?

In Fig 2: both n and N are better to be reported in each step. For example, 9179/9700 (94.6%) were eligible; 5113/9179 (55.7%) of eligibles were contacted; 3654/9179 (39.8%) of eligibles responded. In addition, is there any possibility of adding ranges to each % in each step in this fig 2? If yes, I would also recommend to add them.

Results paragraph 2:

This sentence, “Participation was 71.5% nationally” is not in line with what can be seen from Fig 2. This is true that the conditioning populations are different, but I would recommend having the same numbers in both Table 2 and text. 71.5% was among those contacted, but this is misleading (an appreciably high %). This should be also reported by the total eligibles (which is 39.8%, with range ##, ##).

Results Nonresponse adjustment:

strongest predictors and the beta coefficients were identified and obtained using an unstandardized format of the variable (right?). If yes, this approach cannot identify the strong predictors as each predictor has its own specific unit (not consistent units). To identify the strong predictors, standardized approach should be used. This standardized approach is not typically recommended in health sciences, but for the case of this research and to identify factors that are strongly associated with response to the survey (not based on P-value, rather based on the magnitude of the estimate), standardized coefficients should be used.

“presumed HIV care status” should be better explained and defined as this is the single factor remained in the model to be a strong predictor of response. How “frequency of HIV lab results” was defined? Was it a count variable: 0 result, 1 result, 2 results, 3 results, and so one? What did 0 results indicate and what other results indicated.

Which “model fit statistics” were used to compare both weighting class and propensity model?

The authors chose the cell (class) weighting method “because, having less variance, it performed better than the propensity method.” This means nothing unless there are statistics and some findings to support such a claim. Without these statistics, we cannot evaluate or even trust why class weighting performed better.

The authors believe that class weighting was less variant: a) first less variant requires some quantitative supports, b) how much less was considered to be performing better? This is why I believe details should be reported as the journal does not have any limitation on the number of pages or words.

The whole results and methods sections are bare, meaning that the authors, unfortunately, did not provide details of their approaches to cover their methods, and required statistics and results to support their claim. A detailed explanation and description of the method section as well as detailed provision of results are required.

Results last paragraph:

“produced similar results” requires detailed statistics. What does “similar” mean in this context?

The authors believe that “Because implementing a two-stage adjustment was generally not feasible locally, we opted for a single-stage adjustment nationally”: is this also a new or innovative part of the results section? I am wondering which one of these two (single-stage or two stages) was performed in the previous cycles? As the two-stage was not feasible locally in this new version of the weighting, I think it had also not been feasible previously, right? So, this is not really a surprising result of this manuscript? Please provide explanations on this too.

Discussion and conclusion:

The authors should pay attention to the point that performing multiple weighting steps in surveys is to improve the REPRESENTATIVENESS of the selected sample. Given this, do the authors believe that a two-stage approach (when both noncontact and nonresponse were considered in computing weights) could not provide a more accurate estimate representative to the frame sample than a single-stage? I am wondering who much their findings and suggestions are based on the type of data they use (i.e., a data-driven approach) than the evidence supported by statistical theories (i.e., a theory-supported approach) that having multiple or sequential stages in weighting can better provide representative estimates? This should be also discussed in the discussion section.
