# Supplementary material for: Changes to the sample design and weighting methods of a public health surveillance system to also include persons not receiving HIV medical care
Source: PLoS One. 2020 Dec 3;15(12):e0243351. doi: 10.1371/journal.pone.0243351 (PMC7714102; doi:10.1371/journal.pone.0243351)
Supplement: Attachment — Submitted filename: Comments to the authors PONE-D-19-17946_R1.docx [file pone.0243351.s004.docx]

**Comments to the authors:**

This study is about calculating survey weights, the approach chosen, and the benefits of using a dynamic surveillance registry as a sampling frame, an exciting study. However, many shortcomings should be addressed/implemented.

**Here are my detailed comments with special attention in methodological parts:**

**ABSTRACT**

- **State the design of the study clearly.**
- **State the source of subjects stated.**
- **Present the results using suitable statistical measures with CI's.**
- **State the implications of key findings as conclusions based on the results. The last sentence could not be concluded thru the obtained results.**

**INTRODUCTION**

- **Explain the gap of knowledge and necessity of the study precisely.**

**METHODS**

- **Divide the methods section into determined subsections with defined subheadings according to the STROBE statement.**
- **State the design of the study and its key elements clearly.**
- **State the setting of the study clearly.**
- **State the relevant dates of study, including periods of recruitment and data collection.**
- **State the source of subjects.**
- **State the number of subjects.**
- **Explain the sampling design and procedures in detail.**
- **State the study variables (including outcomes, predictors, and potential confounders).**
- **State the name, version, and address of the software.**
- **Describe the statistical analytical methods taking account of the sampling strategy.**
- **Describe the sensitivity analyses clearly.**
- **Mention the ethics code.**

**RESULTS**

- **Present the characteristics of study participants (e.g., demographic, clinical, and social) and information on exposures and potential confounders.**
- **Present the table of demographic characteristics.**
- **Summarize and describe the outcome measures of the study with suitable statistical measures and their CI.**

**DISCUSSION**

- **Discuss the generalizability (external validity, applicability) of the trial findings that.**
- **Recommend further studies in the future.**
